# Review of Psilocybin Use for Depression among Cancer Patients after Approval in Oregon

**DOI:** 10.3390/cancers16091702

**Published:** 2024-04-27

**Authors:** Val Bellman

**Affiliations:** Psychiatry Residency Training Program, University of Missouri Kansas City, Kansas City, MO 64108, USA; vvzzw8@umkc.edu

**Keywords:** cancer, depression, psilocybin, end-of-life care, Oregon

## Abstract

**Simple Summary:**

Psilocybin therapy shows promise for reducing anxiety and depression and improving psychological well-being in cancer patients nearing the end of life. However, providing this treatment outside of tightly controlled research studies is extremely challenging. In 2020, Oregon became the first U.S. state to legalize psilocybin therapy for depression related to terminal illnesses such as advanced cancer. This review examines the published evidence on psilocybin’s therapeutic potential as well as the multitude of legal, ethical, and logistical hurdles Oregon has faced while attempting to roll out regulated psilocybin services over the past year. The aim is to shed light on the complex issues involved in responsibly implementing psychedelic-assisted therapy for patient populations with serious psychological distress. By proactively identifying and addressing these challenges, appropriate standards of care and equitable access to psilocybin treatment can be ensured as it transitions from research to real-world clinical practice. Insights from Oregon’s experience as the pioneering state can help guide a rigorous, ethical path forward.

**Abstract:**

Despite the legalization of psilocybin therapy for depression in terminal illnesses such as advanced cancer through Oregon’s Measure 109 in 2020, significant challenges have impeded its implementation. This review synthesizes the empirical data supporting the utilization of psilocybin therapy for addressing cancer-related depression, including an evaluation of its purported benefits and potential adverse effects. It provides a comprehensive examination of therapeutic strategies, dosing regimens, and barriers to ensuring responsible and equitable access. Salient issues explored include the development of ethical protocols, integration within healthcare systems, ensuring statewide availability, resolving legal ambiguities, and defining clinical standards. Oregon’s pioneering role serves as a case study, highlighting the necessity of addressing regulatory, logistical, and ethical obstacles to ensure the establishment of rigorous and equitable psilocybin care models.

## 1. Introduction

In November 2020, Oregon became the first U.S. state to legalize the therapeutic use of psilocybin, the active compound found in certain species of fungi. This unprecedented legislation paved the way for Oregon Health & Science University’s (OHSU) psilocybin services program, which began offering psilocybin-assisted therapy in January 2023 under the supervision of licensed facilitators for patients with depression and anxiety related to life-threatening illnesses [1,2]. As researchers continue studying psilocybin as a potential treatment for various mental health conditions, Oregon’s legalization has generated substantial interest in the implementation and outcomes of its psilocybin therapy program.

This article provides a comprehensive assessment of the current landscape surrounding psilocybin’s therapeutic potential within the context of cancer-related depression, highlighting both its promises and challenges in light of Oregon’s recent legislation. By synthesizing existing research, clinical trials, and ethical considerations, this review informs healthcare professionals, policymakers, and the public about the evolving role of psilocybin in addressing the profound psychological toll of cancer and the pursuit of enhanced mental well-being. Furthermore, the legal and ethical considerations of expanding access to psilocybin therapy are discussed, along with key questions still needing investigation regarding safety, efficacy, and best practices for structured psilocybin sessions.

## 2. Depression and Cancer

### 2.1. Exploring the Burden of Depression in the Cancer Population

Depression is a significant concern among cancer patients in the United States, with prevalence rates varying depending on the type of cancer. Studies have consistently shown that the prevalence of depression is higher among cancer patients than in the general population. According to various estimates, the prevalence of depression ranges from 15% to 25% among cancer patients, depending on the type and stage of cancer as well as other factors [3,4]. Studies have shown that the prevalence of depression can range from 3% in patients with lung cancer to as high as 31% in patients with cancer of the digestive tract [4,5]. 

Studying the prevalence of depression in cancer patients is important for several reasons. First, understanding the prevalence and risk factors allows for early detection and intervention, enabling healthcare professionals to identify and support patients at a higher risk of depression [6]. Despite the high prevalence of depression among cancer patients, it often goes undiagnosed and undertreated. Healthcare professionals may overlook the psychological aspects of cancer care, focusing primarily on the physical aspects of treatment [7]. While cancer can increase the risk of depression due to the psychological and physical burden of the disease, depression itself may also increase the risk of developing certain types of cancer, potentially due to the physiological effects of chronic stress and inflammation [8]. It can exacerbate physical symptoms, reduce treatment adherence, and negatively affect treatment outcomes [9]. Several studies have suggested that depression in cancer patients is associated with an increased risk of mortality. This may be due to various factors, including reduced treatment adherence, physiological effects of depression on the immune system, and a higher risk of suicide [10]. Additionally, depression in cancer patients is associated with increased healthcare costs and longer hospital stays [11].

Recognizing demoralization as a separate entity from depression and identifying its unique impact on patients’ well-being can lead to more targeted interventions and improved outcomes. Studies have shown that demoralization is prevalent in this population, with rates ranging from 13% to 50% [12]. Demoralization often coexists with depression, anxiety, adjustment disorders, and suicidal ideation in cancer patients [12,13,14]. While demoralization and depression share similarities, they are distinct conditions, with demoralization characterized by feelings of subjective incompetence and a lack of hope [15,16]. Studies have shown that demoralization can impact various aspects of a cancer patient’s life, including quality of life, sleep quality, spiritual interests, and suicide risk [17,18,19]. Furthermore, demoralization has been linked to factors such as existential anxiety, masculine self-esteem, resilience, and perceived stress, all of which play a role in predicting psychological well-being and the development of depression in cancer patients [20,21,22,23]. It is essential to differentiate demoralization from depression, as some patients may experience demoralization without depression, and vice versa [16,24]. Moreover, demoralization has been identified as a risk factor for suicidal behavior, emphasizing the importance of addressing this condition in cancer care [13,25]. 

The effective management of depression may also lead to better treatment adherence and potentially improved survival rates. Additionally, addressing depression can significantly improve cancer patients’ overall quality of life by reducing their physical and emotional distress and improving their ability to cope with the challenges of treatment [26,27]. Early identification and interventions targeting demoralization, along with hopelessness and depression, are crucial in preventing the development of suicidal ideation in cancer patients [14,28]. Finally, understanding the scope of the problem can inform research efforts and guide the allocation of resources for developing effective screening, treatment, and support strategies for cancer patients with depression.

### 2.2. Psychiatric Treatment of Depression in Cancer Patients

Psychopharmacological treatment for cancer-related depression is a complex area that requires a tailored approach to meet the individual needs of patients. Research indicates that both psychosocial interventions and pharmacotherapy are effective in treating depression in cancer patients [29]. Antidepressants are frequently prescribed to cancer patients with depression, but studies have shown that they have limited effectiveness in improving depressive symptoms in terminally ill cancer patients [29,30]. While pharmacological treatment is important in managing depression, especially in advanced cancer patients, the characteristics of these patients can influence the choice of pharmacological interventions [31]. Additionally, the pharmacological treatment of depression in cancer patients is complicated by drug–drug interactions between antidepressants and cancer pharmacotherapy, potentially reducing the efficacy of cancer treatment [32,33]. Despite the widespread use of antidepressants in cancer care, their effectiveness remains a subject of debate, with some studies indicating that currently prescribed antidepressants may only be efficacious in a limited subset of patients [34]. Additionally, the use of conventional antidepressants in cancer patients has been linked to decreased compliance with anticancer treatments [35]. Table 1 provides additional details regarding pharmacological interventions for depression in cancer patients. 

Studies have suggested that combined pharmacological and psychological treatments may be more effective than either approach alone in primary depression [38]. However, this combined approach has not been extensively tested in cancer populations, indicating a gap in knowledge that requires further investigation [38]. Additionally, recent literature reviews have provided mixed evidence regarding the effectiveness of antidepressant drugs for depression in cancer patients [39,40].

The critical need for a novel drug for depression in cancer patients arises from the limitations of current antidepressants; the unique features and challenges of cancer-related depression; the potential to improve quality of life, treatment adherence, and overall outcomes; and the need for tailored approaches for specific cancer populations [41]. Dysregulated pathways implicated in cancer-induced depression could be addressed by novel therapeutics to alleviate depressive symptoms in these patients [42]. Additionally, drug repositioning approaches focusing on immune alterations and inflammation have demonstrated the potential for novel pharmacological interventions in depression by targeting inflammatory mechanisms [43]. The emergence of new drugs for treating major depression, such as curcumin, provides a hopeful avenue for addressing depression in cancer patients [44]. Novel drug discovery efforts, such as exploring traditional Chinese medicine compounds like oxymatrine, have been suggested as a strategy to develop effective therapies for cancer patients, aiming to enhance treatment efficacy and overcome drug resistance [45].

## 3. Role of Psilocybin in the Treatment of Depression

### 3.1. Background on Psilocybin and Its Antidepressant Effects

Psilocybin, a hallucinogenic compound found in certain mushrooms, has garnered attention for its antidepressant effects, particularly in the context of life-threatening diseases such as cancer. Research has shown promising results in using psilocybin to alleviate psychological distress in cancer patients [46,47,48,49,50]. Studies have shown that psilocybin’s antidepressant effects may not necessarily rely on altered perception, suggesting that its mechanism of action in treating depression can be studied independently [51]. Neuroimaging studies in healthy volunteers have revealed that psilocybin produces profound and meaningful alterations in brain function, especially of the default mode network, consistent with an antidepressant effect [52]. Furthermore, investigations into the neural correlates of psilocybin administration have revealed changes in brain activity and connectivity, indicating a putative neural mechanism of action for psilocybin [53,54]. Other studies have also indicated increased global integration in the brain after psilocybin therapy for depression, suggesting potential neuromodulatory effects [55,56]. Psilocybin therapy has also been shown to increase cognitive flexibility for at least four weeks post-treatment [57]. This revival of emotional responsiveness on a neural and psychological level is proposed as a key treatment mechanism for psychedelic therapy. 

Psilocin, the active metabolite of psilocybin, has been shown to increase the concentrations of dopamine and serotonin in specific brain pathways [58,59]. These compounds are structurally similar to serotonin, with slight chemical modifications that lead to hallucinogenic effects [60]. Psilocybin is dephosphorylated in vivo to form psilocin, which is responsible for its psychoactive effects [59]. Psilocin is metabolized through oxidative deamination to form 4-hydroxy-3-indoleacetic acid and psilocin-O-glucuronide [61].

**Figure 1:**
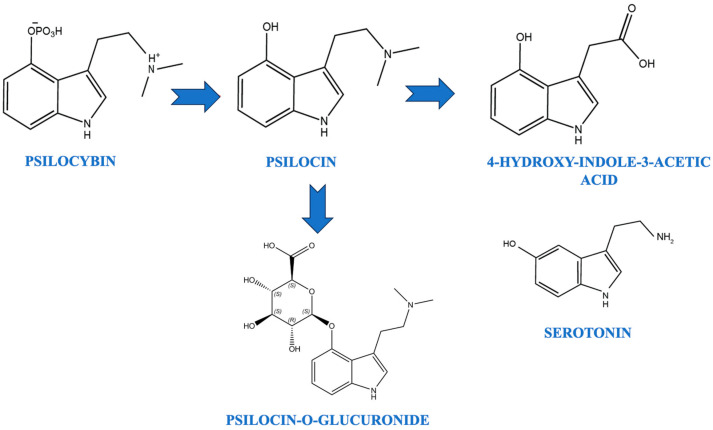
Pharmacological conversion of psilocybin to psilocin.

The 4-hydroxyl group of psilocin likely contributes to its oral bioavailability and enhanced metabolic stability, aiding in improved central nervous system penetration [62]. The levels of 5-hydroxyindoleacetic acid (5-HIAA) in relation to serotonin were found to be elevated in specific brain regions such as the hypothalamus and pons [63]. Psilocin easily crosses the blood–brain barrier and exerts its psychoactive effects by acting on serotonin receptors [64]. Figure 1 summarizes the pharmacological conversion of psilocybin to psilocin. 

The overall psychedelic experience induced by psilocybin results from a complex interplay between various serotonin receptor subtypes, as well as interactions with other neurotransmitter systems and brain regions. Psilocybin exerts its effects primarily through agonist activity at the 5-HT2A receptor [48,65,66,67,68]. The activation of the 5-HT2A receptor by psilocybin has been associated with various outcomes, including visual hallucinations, increased cortical excitability, and alterations in visual-evoked cortical responses [65]. Psilocybin’s stimulation of 5-HT2A/1A receptors has been linked to a reduction in social pain processing and the induction of long-term changes in mindfulness [69,70]. Studies have shown that psilocybin treatment can lead to increased global integration in the brain, with higher-order functional networks becoming more interconnected and flexible [55]. Furthermore, research suggests that the behavioral and synaptic actions of psilocybin may be independent of 5-HT2A receptor activation, as evidenced by studies in which responses to psilocybin were not prevented by a 5-HT2A/2C antagonist [51]. Psilocybin’s effects on brain dynamics have been associated with the distribution of 5-HT2A receptors across the cortex, influencing brain control energy landscapes [71]. Additionally, psilocybin has been shown to activate both 5-HT1A and 5-HT2A receptor subtypes, impacting attention, working memory, and other cognitive functions [72]. Table 2 provides additional data regarding serotonergic receptors affected by psilocybin. 

### 3.2. Psilocybin for End-of-Life Care and Cancer-Related Depression

Psychedelic therapy, particularly with compounds like psilocybin, has shown promising potential in treating cancer-related depression and psychological distress. Most studies have been conducted in controlled clinical settings, typically involving preparatory sessions, the administration of psilocybin under supervision, and integration sessions afterwards. Participants were generally patients with advanced or terminal cancer diagnoses experiencing anxiety, depression, or existential distress related to their condition [75,76,77,78]. For example, a pilot study at the University of California, Los Angeles, reignited interest in psilocybin treatment for advanced-stage cancer patients, leading to renewed efforts in psilocybin research [58]. Furthermore, the FDA granted the breakthrough therapy designation for psilocybin in the treatment of depression, indicating its potential as a novel therapeutic approach [79,80]. 

Clinical trials have demonstrated significant reductions in anxiety and depression in cancer patients following psilocybin treatment, with sustained symptom reduction observed [46,47,81,82]. The psychedelic experience, combined with psychological support, appears to help alleviate the mental health burdens often associated with cancer diagnosis and treatment. Psilocybin has been found to produce rapid, notable, and lasting effects, leading to immediate, substantial, and sustained improvements in anxiety and depression; decreases in cancer-related demoralization and hopelessness; and sustained benefits in existential distress [83,84,85,86]. It has also been associated with improvements in feelings of connectedness and acceptance, quality of life, and attitudes toward death [87,88].

Long-term follow-up studies have documented sustained improvements in anxiety and depression, as well as improved quality of life, in cancer patients following psilocybin treatment [82]. These positive effects were often sustained for several months after the psilocybin sessions [89,90,91]. The legalization of psilocybin therapy in certain regions, such as Oregon, in the United States, and Canada, reflects the growing acceptance and recognition of its potential therapeutic benefits [92]. 

There is increasing interest in understanding the negative outcomes associated with psilocybin use [93]. Research indicates that psilocybin administration can lead to effects such as silliness, laughter, and playfulness, which may be perceived as unconventional but can be modulated to align with the desired clinical outcomes [94]. It is important to note that psilocybin has potent psychedelic properties and is illegal in most countries, with reports of serious negative health-related outcomes in non-clinical settings due to poorly regulated behavior [95,96,97]. Studies have found that younger individuals are more likely to require emergency medical treatment after using psilocybin than older individuals. The most frequently reported adverse reactions are psychological in nature, such as severe anxiety, panic attacks, paranoia, and psychotic symptoms [98]. Physiological side effects such as headaches, dizziness, gastrointestinal distress, and changes in blood pressure are also possible when taking psilocybin [99]. The primary reasons cited for adverse incidents are an unprepared or negative psychological state prior to the psilocybin experience, an unsuitable or unsupportive physical environment or setting, and the mixing of psilocybin with other substances or drugs [98].

### 3.3. Risk of Combining Psilocybin and Traditional Antidepressants in Cancer Patients

Combining psilocybin with traditional antidepressants in cancer patients raises concerns due to potential interactions and safety issues. While psilocybin has shown promise in enhancing the well-being of cancer patients [100], its combination with antidepressants may have varying effects. The main concern with combining psilocybin and antidepressant medications is the risk of developing serotonin syndrome. Additionally, serotonergic antidepressant use has been associated with weaker psilocybin effects and potential adverse reactions [89]. Certain antidepressants like MAOIs are generally contraindicated with psilocybin due to the risk of hazardous interactions [101]. Moreover, abruptly stopping antidepressants before psilocybin use can trigger discontinuation symptoms [102]. Table 3 summarizes these drug–drug interactions. 

## 4. Psilocybin in Oregon

The state of Oregon has taken a progressive stance on psilocybin therapy by legalizing it through Oregon Measure 109, the Psilocybin Mushroom Services Program Initiative [92]. This initiative allows adults aged 21 years and older to take psilocybin under supervision in state-licensed service centers, making Oregon the first state in the United States to decriminalize the use of psilocybin-containing mushrooms [113]. Psilocybin-assisted therapy has demonstrated rapid-acting and persisting antidepressant effects from just one or two doses, as evidenced by early phase studies [86]. This suggests that Oregon’s Measure 109 aligns with the growing body of research supporting the potential therapeutic benefits of psilocybin in the treatment of psychiatric disorders. Despite the passage of the law, psilocybin continues to be prohibited under federal law unless it is rescheduled. Until such rescheduling takes place, there is a possibility of a form of “cooperative federalism” emerging with regard to psilocybin use in Oregon [82]. 

The Oregon Psilocybin Services Act mandates that the Oregon Health Authority (OHA) oversee the licensing, manufacturing, transportation, delivery, sale, and purchase of psilocybin products, creating a structured framework for the provision of psilocybin services within medical care settings [53]. The Act aims to establish a system of licensing, training, and tracking to ensure the safe and responsible delivery of psilocybin services [114]. It specifies that advertising psilocybin products and services is generally prohibited, along with importing, transporting, distributing, or possessing psilocybin products without the proper licensing. It does not allow for retail sale. Additionally, the Act requires psychological support throughout psilocybin therapy to ensure the safety and well-being of participants [115]. Table 4 outlines some key aspects of the regulatory oversight established under this Act. 

Oregon’s Measure 109 reflects a shift toward embracing the potential of psilocybin as a therapeutic agent, drawing from the historical use of psilocybin and modern medical research [116]. The crux is the regulatory and ethical complexities of implementing a treatment modality involving an illegal substance in a non-medicalized framework while still ensuring appropriate oversight, training, and access. Individuals with licenses are immune to Oregon’s criminal statutes concerning the possession, distribution, or production of psilocybin, as well as aiding and abetting others in these activities, or any other criminal act where psilocybin possession, distribution, or production is involved [117]. The proposed measure enables terminally ill patients suffering from anxiety and depression to avail themselves of psilocybin therapy. However, an Oregon law provision also enabled counties and cities to vote against permitting psilocybin services within their boundaries after the statewide measure was approved [118]. Numerous counties and municipalities across Oregon exercised this option to prohibit psilocybin services, resulting in substantial areas of the state opting out of the voter-approved program [119]. This severely limits access to psilocybin therapy for cancer patients in those opt-out areas, depriving them of a potential treatment option to help cope with the psychological distress and anxiety often experienced during cancer treatment [120]. Moreover, practical implementation for terminally ill individuals also faces regulatory hurdles, systemic barriers, clinical reservations, and a lack of specialized training and oversight protocols [121,122,123]. Table 5 outlines some potential issues that could limit the use of psilocybin for terminally ill individuals, despite initial state approval.

Although the approval of psilocybin presents promising therapeutic opportunities, it is beset by challenges related to inadequate supply and availability, while terminally ill patients cannot afford to delay access [143]. This psilocybin program allows the use of *Psilocybe cubensis*, despite there being over 100 other types of psilocybin-producing fungi [144]. Drawing a parallel with the cannabis industry, which offers various strains catering to different needs [145], the researchers argue that different strains of psilocybin-producing fungi may offer different benefits [146,147,148]. Unfortunately, Oregon’s current rules may overlook the potential benefits of other psilocybin varieties [81,147]. According to the Right to Try (RTT) framework, the therapeutic use of psilocybin for patients with life-threatening illnesses or conditions is protected, as psilocybin qualifies as an “eligible investigational drug”, having completed Phase I clinical trials while still under investigation [149]. Prior to the implementation of Oregon’s Psilocybin Services Act (PSA), terminally ill individuals in Oregon struggling with anxiety and/or depression were legally able to access psilocybin therapy, although supporting data on this pathway are unavailable [150,151].

This narrative review’s scope is restricted by the quality and features of the currently available published research examining the use of psilocybin for alleviating psychological distress in individuals with cancer. While randomized trials were included, sample sizes were often small, and the study populations lacked diversity across racial/ethnic, gender, and socioeconomic factors. There are minimal data on psilocybin therapy outside of life-threatening cancer diagnoses or comparing its efficacy to existing interventions. Significant uncertainty remains regarding optimal dosing strategies, long-term safety, delivery methods beyond oral administration, and the neurobiological mechanisms underlying therapeutic effects. Furthermore, the logistical and systemic challenges surrounding the equitable implementation and healthcare integration of psilocybin therapy in real-world settings have not been well characterized. Despite the comprehensive nature of this review, the interpretability and generalizability of the findings are constrained by the limitations of the primary evidence base. Continued rigorous research involving larger, more diverse clinical trials is needed to address these limitations.

## 5. Conclusions

The emerging body of evidence supports the potential therapeutic benefits of psilocybin for cancer-related depression and anxiety. Multiple clinical trials have demonstrated rapid and sustained reductions in depression, anxiety, demoralization, and existential distress among cancer patients treated with psilocybin, along with improved quality of life and attitudes toward death. Neural imaging studies point to putative mechanisms involving increased brain network integration and connectivity following psilocybin therapy. 

As the first state to legalize psilocybin services, Oregon’s initiatives reflect this promise but have also exposed challenges in practically implementing psilocybin therapy outside of tightly controlled research settings. Legal ambiguities, social stigma, lack of professional integration pathways, and limited regulatory guidance could hinder appropriate patient access and standards of care. Key considerations include training requirements for facilitators, ethical administration protocols, equitable availability regardless of geography, and avenues for rigorous data collection to assess safety and outcomes. Further research is still needed on optimal therapeutic approaches, coordination with existing cancer care, dosing strategies, long-term effects, and suitability for specific patient populations. Addressing knowledge gaps through careful study and open dialogue among stakeholders will be critical as psilocybin therapy transitions from empirical investigation to approved clinical practice. Despite the hurdles ahead, Oregon’s pioneering framework offers invaluable lessons for establishing comprehensive psychedelic medicine programs focused on patient well-being.

## Figures and Tables

**Table 1:** Targeted psychopharmacological treatment for depression in cancer patients.

Medication	Comment
Bupropion	Beneficial for alleviating fatigue and indifference. Beneficial for individuals seeking to quit smoking. When to avoid: anxiety, brain tumors, risk of alcohol withdrawal [36].
Fluoxetine	Appropriate for patients who are NPO (nothing by mouth) or with intermittent bowel obstruction. The longest-acting SSRI [36].
SNRI	Beneficial for individuals experiencing concurrent neuropathy. A viable option for pain that is not fully alleviated by opioid medication [36].
Venlafaxine	Primary choice for patients with breast cancer using tamoxifen. Lacks inhibition of 2D6 enzymes. Suitable for concurrent use with other medications.
Duloxetine	Should not be used in individuals with liver or kidney impairment [37].
Levomilnacipran	Only approved for fibromyalgia.
Mirtazapine	Can be given as a sublingual or orally dissolving tablet. Beneficial for individuals having trouble swallowing. Can aid sleep at doses of 7.5–15 mg per day. May assist in reducing nausea and increasing appetite [36].
Tricyclic Antidepressants	Not typically the initial treatment option. Used for neuropathy or persistent headaches.
Methylphenidate	Given at doses of 2.5–10 mg. Used for individuals experiencing profound depression and those with extremely low energy levels. Used for patients unable to endure the typical 3–4 week waiting period for SSRI/SNRI effectiveness [36].
Dextroamphetamines	Given to terminally ill patients with persistent fatigue. Used for patients unable to endure the typical 3–4 week waiting period for SSRI/SNRI effectiveness [36].
Modafinil	Used as a second line. The cost of this medication can be a barrier for patients.

**Table 2:** Psilocybin and serotonin receptors.

Receptor	Location	Targets	Comment(s)
5-HT1AR	CNS: high density in cerebral cortex, hippocampus, septum, amygdala, and raphe nucleus; small amounts in basal ganglia and thalamus	CNS: aggression, anxiety, appetite, memory, mood CVS: vasoconstriction, BP, HR	Psilocybin has a moderate affinity for the 5-HT1A receptor, which is involved in regulating serotonin release and neuronal excitability [69,70,73].
5-HT2AR	CNS: basal ganglia and other structures	CNS: anxiety, imagination, learning, perception SM: contraction Platelet: aggregation	Activation of 5-HT2A is thought to be responsible for profound alterations in perception, mood, and cognition. Other effects include visual distortions, altered perceptions of time and space, and changes in thought patterns [65,66,67,68,73,74].
5-HT2CR	CNS: hippocampus and substantia nigra	CNS: mood, sleep, anxiety	Lower potency than the 5-HT2A receptor. Activation of the 5-HT2C receptor contributes to the emotional and cognitive effects of psilocybin, such as altered mood states and increased introspection [51,73,74].

Abbreviations: 5-HT: 5-Hydroxytryptamine (serotonin). 5-HT1AR: 5-Hydroxytryptamine (serotonin) 1A Receptor. 5-HT2AR: 5-Hydroxytryptamine (serotonin) 2A Receptor. 5-HT2CR: 5-Hydroxytryptamine (serotonin) 2C Receptor.

**Table 3:** Interaction between psilocybin and antidepressants.

Medications	Psilocybin Use
SSRI/SNRI	Consider tapering and discontinuing antidepressant medication at least two weeks before psilocybin use (except for fluoxetine, which necessitates a six-week interval) to mitigate potential diminishment of the psychedelic effect. Prolonged antidepressant use might lead to the down-regulation of 5HT2A receptors, resulting in diminished psychedelic experiences for some individuals [89,103,104,105].
Bupropion	The loss of effect is not predicted to occur; consider tapering and discontinuation prior to psychedelic use [105].
Mirtazapine	Taper and discontinue 2+ weeks prior to avoid loss of psychedelic effect. Mirtazapine blocks the 5H2A receptor, which is predicted to result in blunting or loss of psychedelic effects [106].
Tricyclic Antidepressants (TCA)	Consider tapering and discontinuing at least 2 weeks prior due to potential intensified effects [107].
Trazodone	Taper and discontinue at least 5 days prior due to the potential loss of psychedelic effect. Trazodone, at lower doses (25–150 mg), blocks 5HT2A receptors and begins to block the serotonin reuptake pump at doses exceeding 150 mg [108,109]. It also possesses an active metabolite that blocks 5HT2A receptors and modulates numerous other 5HT receptors.
Buspirone	Gradually reduce and stop the medication at least five days before to mitigate the risk of diminished psychedelic effects. Buspirone functions as a non-psychedelic partial activator at serotonin receptors, potentially resulting in reduced psychedelic effects [110]. Minimal risk of serotonin syndrome
MAO-A Inhibitors *	Consider tapering and discontinuing the medication two weeks before to prevent diminished psychedelic effects. Risk of cardiovascular collapse [111].
MAO-B Inhibitors	Psilocybin combination poses low physical toxicity risks [112]

* chronic use.

**Table 4:** Aspects of comprehensive regulatory framework for psilocybin products and psilocybin services in Oregon.

Licensing and registration	The OHA is tasked with developing a licensing and registration program for psilocybin product manufacturers, facilitators, service centers, and testing laboratories. Facilities and facilitators must meet the training and professional requirements set by the OHA.
Role of service centers	Only licensed service centers can provide psilocybin services. Facilitators must be present at all times during the administration session. There are strict eligibility and screening requirements for those seeking access to psilocybin services. There will be a two-year development period to allow the OHA time to establish regulations before licensed facilities can open. Facilities can only purchase psilocybin products from licensed manufacturers.
Oversight	The Oregon Psilocybin Advisory Board is to advise the OHA and the Psilocybin Control Board, which adopts rules related to possession, transportation, delivery, sale, and purchase of psilocybin products.

**Table 5:** Barriers to psilocybin access for terminally ill patients despite state legalization.

Reason	Details
Concerns about public health and safety	Fear of increased substance abuse and misuse. Potential for adverse effects on mental health, especially in vulnerable populations [118,124].
Social and cultural stigma surrounding psychedelics	Longstanding negative perceptions of psychedelic drugs in mainstream society. Reluctance to embrace alternative forms of therapy and treatment [118,124].
Lack of education and awareness in general public	Misunderstandings about the therapeutic potential of psilocybin. Limited knowledge about scientific research supporting its efficacy in treating certain mental health conditions.
Healthcare system integration	Avoidance of medicalizing the practice, referring to “clients” not “patients,” and prohibiting the term “therapy” [124], which contrasts with the original intent of providing a therapeutic treatment. The supported adult use of psilocybin is legally barred from being considered therapy or medical treatment. Any facilities providing psilocybin services are legally prohibited from being situated inside or affiliated with healthcare facilities [125,126].
Regulatory challenges	Uncertainty about how to effectively regulate psilocybin treatment centers.
Professional licensing	It is unclear whether major professional organizations will endorse or accept psilocybin-assisted therapy as a legitimate intervention [115,127]. Concerns about ensuring vulnerable individuals can access licensed mental health professionals, not just non-medical facilitators. For those acting as psilocybin facilitators, if they hold professional licenses in fields such as nursing or medicine, they are not permitted to utilize the scope of practice granted by those licenses when providing psilocybin services [128]. Challenges for licensed providers in adapting to a non-therapy model when state rules explicitly prohibit “performing therapy” with psilocybin [129].
Insurance coverage	Insurance coverage remains a major barrier. It may become required if psilocybin gains FDA approval, though insurers could still restrict or limit coverage, impacting affordability and access [130].
Liability	Liability insurance providers may be hesitant to offer coverage for professionals conducting this type of therapy due to the legal status of psilocybin [131,132,133,134]. Ambiguity around which licensing bodies would handle complaints or discipline for psilocybin facilitators [135].
Moral and ethical considerations	Ethical dilemmas around having clients/patients participate in a treatment that involves an illegal substance at the federal level [136,137].
Informed consent	Current processes may not fully cover issues like suggestibility, power dynamics, and potential adverse effects during psilocybin sessions [138]. The mandatory consent form clients must sign explicitly states that the psilocybin services they are receiving “are not considered a medical or clinical treatment” [139].
Limited administration methods	May exclude individuals with serious medical conditions like cancer who cannot ingest psilocybin orally due to issues such as muscle weakness or difficulty swallowing. The OHA has interpreted the term “consume” to mean “orally ingest”, thus restricting the options available for psilocybin administration [140].
Research	Ongoing research is still needed to determine which conditions benefit most and what are the safest, most effective administration methods [141]. Measure 109 lacks clear guidelines for reporting metrics to assess the safety, fairness, or effectiveness of psilocybin services [142].
